# Organic matter cycling in a model restored wetland receiving complex effluent

**DOI:** 10.1007/s10533-022-01002-x

**Published:** 2022-12-11

**Authors:** Xingzi Zhou, Sarah Ellen Johnston, Matthew J. Bogard

**Affiliations:** 1 Department of Biological Sciences, University of Lethbridge, Lethbridge, AB Canada; 2 Present Address: Department of Chemistry and Biochemistry, University of Alaska Fairbanks, Fairbanks, AK USA

**Keywords:** Effluent, Wetland, Dissolved organic matter (DOM), PARAFAC, Mass balance, Water residence time (WRT)

## Abstract

Wetlands have been used to treat anthropogenic effluents for decades due to their intense biogeochemical processes that transform and uptake nutrients, organic matter, and toxins. Despite these known functions, we lack generalizable knowledge of effluent-derived dissolved organic matter (DOM) cycling in wetlands. Here, we quantify the cycling of DOM in one of Canada’s more economically important wetland complexes (Frank Lake, Alberta), restored to hydrologic permanence in the 1980s using urban and agro-industrial effluents. Optical analyses and PARAFAC (parallel factor analysis) modelling showed a clear compositional change from more bioavailable and protein-like DOM at effluent input sites to more aromatic and humic-like at the wetland outflow, likely due to DOM processing and inputs from marsh plants and wetland soils. Microbial incubations showed that effluent DOM was rapidly consumed, with the half-life of DOM increasing from as low as 35 days for effluent, to 462 days at the outflow, as a function of compositional shifts toward aromatic, humic-like material. Long-term averaged dissolved organic carbon (DOC) export was low compared to many wetlands (10.3 ± 2.0 g C m^−2^ yr^−1^). Consistent with predictions based on water residence time, our mass balance showed Frank Lake was a net source of DOM across all measured years, but shifted from a source to sink among wet and drought years that respectively shortened or lengthened the water residence and DOM processing times. Overall, Frank Lake processes and transforms effluent DOM, despite being a longer-term net source of DOM to downstream environments.

**Supplementary Information:**

The online version contains supplementary material available at 10.1007/s10533-022-01002-x.

## Introduction

Wetlands provide many economically valuable services including the conservation of biodiversity, flood water storage and management, and water treatment (Euliss et al. 2008; Mitsch and Gosselink 2000). Natural and constructed wetlands (both considered as treatment wetlands here) have been used worldwide for decades in the processing of anthropogenic effluents (Euliss et al. 2008; Werker et al. 2002). Intense biogeochemical cycling in wetland sediments, littoral vegetation, and by suspended plankton (microbes, phytoplankton) can make wetlands efficient sites for the processing of organic matter (OM) and nutrients (Werker et al. 2002). Previous reviews indicate that globally, both natural (Fisher and Acreman (2004)) and constructed wetlands (Vymazal 2007) are often strong nitrogen (N) and phosphorus (P) sinks. Yet for dissolved organic matter (DOM), net processing (production versus consumption) can vary with the source and composition of DOM inputs, as well as wetland conditions (vegetation type, hydrology, soil type, temperature, and pH, etc.), as shown for both constructed and natural wetlands in the U.S. (Lu et al. 2003; Pinney et al. 2000). Studies from around the world from natural and experimental systems have shown that the net outcome of DOM cycling also depends on processes including microbial mineralization and photodegradation of DOM (Hertkorn et al. 2016; Li et al. 2008; Stottmeister et al. 2003). Further, anaerobic conditions in wetland sediments can restrict the efficiency of DOM mineralization, but can also enhance removal through processes such as denitrification (Kayranli et al. 2010; Werker et al. 2002), sedimentation, and sorption (Fisher and Acreman 2004; Vymazal 2007). Taken together, wetland restoration is a potential mechanism to improve water quality through increased inorganic nutrient processing (Cheng et al. 2020), yet the role of wetlands, especially treatment wetlands, in net OM cycling and remediation is not clear.

Overall, DOM represents a complex mixture of organic molecules including macro- and micro-nutrients that support both auto- and heterotrophic activities, which can influence physical and chemical properties in aquatic ecosystems (Findlay and Sinsabaugh 2003). Broadly-speaking, DOM is categorized into two types: autochthonous (internally-generated in an ecosystem) and allochthonous (externally-derived) DOM. Internally-derived, autochthonous DOM is generally less aromatic (more aliphatic), with lower molecular weight (LMW) and greater bioavailability (Findlay and Sinsabaugh 2003). Natural allochthonous DOM (e.g., from terrestrial soils and forests) is transported into aquatic ecosystems and contains more aromatic, complex compounds (high molecular weight DOM, HMW) with higher C to N ratios, that tend to be less bioavailable (Findlay and Sinsabaugh 2003; van den Berg et al. 2012). Terrestrial DOM inputs provide a large diversity of DOM compounds that vary with vegetation type (Camino-Serrano et al. 2014; Thieme et al. 2019), soil type (Tank et al. 2018; van den Berg et al. 2012), exposure to bio-and photo-degradation processes (Hernes and Benner 2003), residence time on land (Fellman et al. 2013), and hydrological characteristics (Kaiser and Kalbitz 2012; Singh et al. 2014; Tank et al. 2018). A large fraction of this DOM is resistant to microbial consumption over shorter (monthly to annual) timescales and can accumulate through time and space in aquatic networks (Guillemette and del Giorgio 2011; Nebbioso and Piccolo 2013). These processes interact to shape the diversity of DOM and its chemical properties and bioavailability (Camino-Serrano et al. 2014; Singh et al. 2014; Thieme et al. 2019). Therefore, changes to inputs of DOM from individual sources will impact the composition of DOM and may affect the functioning of aquatic ecosystems.

The capacity for wetlands to process DOM is variable and hard to predict, and is affected by diverse human activities that include riparian land use/land disturbance, nutrient inputs from agricultural land, urbanization, and wastewater release (Xenopoulos et al. 2021). Allochthonous OM inputs are enhanced by effluent loading and soil disturbance and erosion by agricultural and other land uses (Regnier et al. 2013; Xenopoulos et al. 2021). Conversely, as shown for marine and inland ecosystems alike, nutrient pollution stimulates autochthonous DOM production via aquatic primary producers (Seitzinger et al. 2002; Xenopoulos et al. 2021). Taken together, human pressures directly and indirectly influence the quality and quantity of DOM through allochthonous and autochthonous pathways. It is therefore not surprising that previous studies have found wide-ranging outcomes in DOM cycling among treatment wetlands. For constructed wetlands in Oregon and California, U.S.A., distinct processing of individual wastewater sources (e.g., industrial, municipal, or livestock waste), plus different input rates of DOM from emergent vegetation and phytoplankton lead to a wide range of outcomes in DOM processing, spanning net DOM accumulation to removal from in- to outflows of individual systems (Barber et al. 2001). The processing of treated urban wastewater in sequential constructed wetland basins in China led to the removal of protein-like DOM and a qualitative shift towards more humic-like DOM from the inlet to outflow, likely due to wetland plant and soil inputs (Yao et al. 2016). Conversely, in a study of constructed wetlands receiving urban stormwater runoff in southern California, three of four systems were net sinks of DOM, despite little change in optical properties from inlets to outlets (Clark et al. 2020). These differences in wetland DOM processing from one ecosystem to the next can have ramifications for society, in part because the concentration and characteristics of DOM can influence the development of disinfection by-products (DBPs) after chlorination in drinking water treatment plants, which can be toxic or carcinogenic to humans (Krasner 2009; Xu et al. 2021). The composition of DOM also impacts the potential toxicity of water, since DOM has high cation exchange capacity which increases the affinity of molecules to adhere to DOM, including metals and contaminants such as pesticides (Supowit et al. 2016; Wood et al. 2011). Given the multitude of factors that regulate DOM cycling in wetlands, it remains difficult to predict how individual wetlands, whether natural or constructed, cycle and transform effluent DOM, and what effect this has on the net production or consumption of DOM at the ecosystem scale. Elucidating the patterns of wetland DOM processing has important consequences for watershed health and water resource management.

**Fig. 1:**
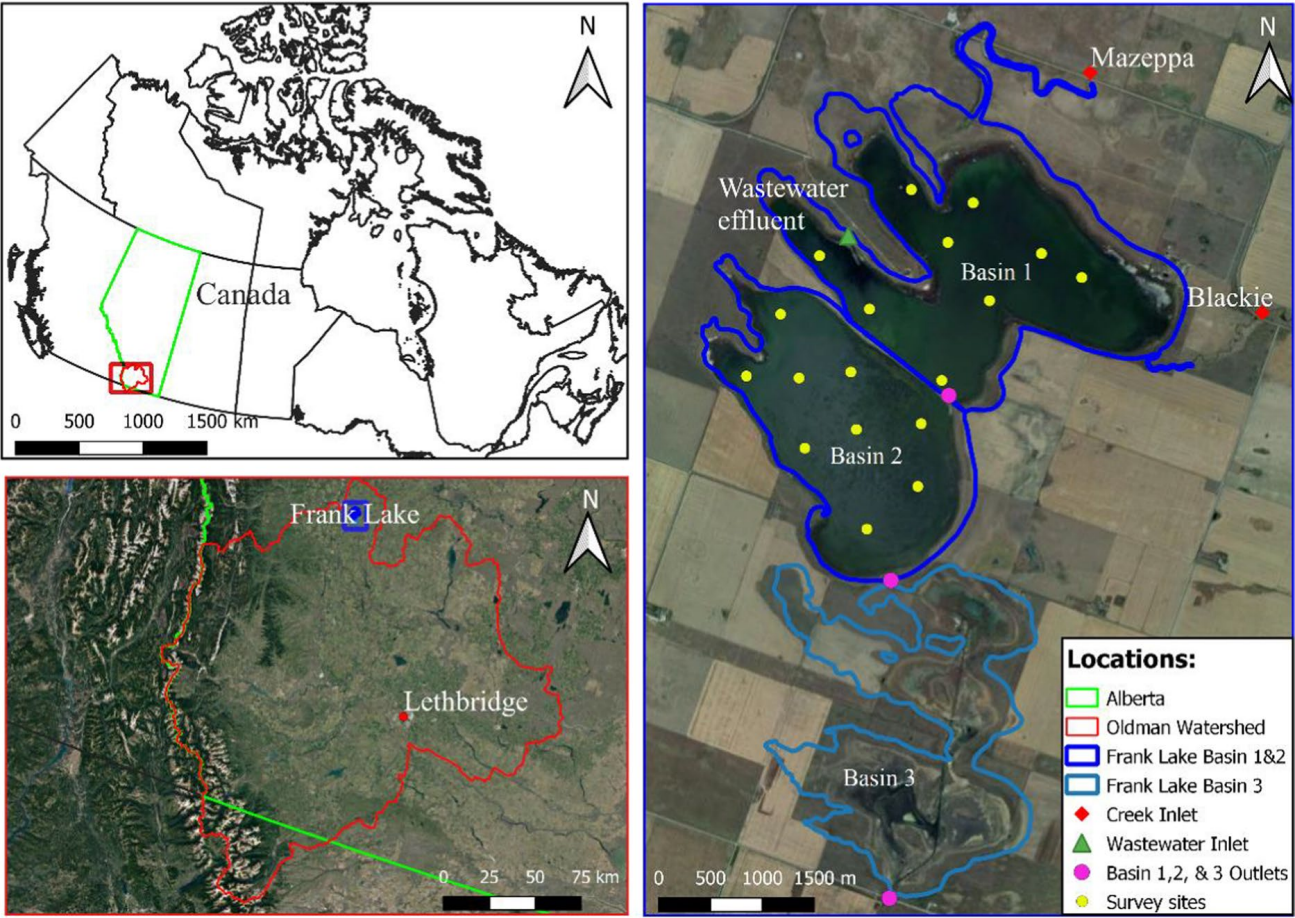
Location of Frank Lake in the Oldman Watershed, Alberta, Canada. Top left: the Oldman Watershed (red outline; ~ 27,500 km^2^) in Canada. Bottom left: study site in the Oldman Watershed (blue box). Right: sampling site for inlets including Blackie (BL) and Mazeppa (MA) creeks (red diamonds) and wastewater effluent (EF; green triangle) and Frank Lake Basin 1, 2, and 3 outflow sites (B1O, B2O, B3O; pink circles) and Basin 1 and 2 survey sites (B1, B2; yellow circles). Google satellite reference: EPSG:3857‐WGS 84/Pseudo-Mercator—Projected

Here, our goal was to define how DOM is cycled in one of Canada’s largest mineral wetland complexes located in the semi-arid northern Great Plains region (Fig. 1), which was restored to hydrologic permanence using effluent inputs from municipal and agro-industrial sources (White and Bayley 1999). To achieve our goal, we aimed to address the following questions: (1) What is the compositional change in the DOM pool along the hydrologic continuum from distinct inputs (effluent and tributaries) to the outflow below the wetland complex? (2) Does the capacity of microbial DOM processing shift in a predictable way along the hydrologic continuum from sources to outflow? (3) Is the wetland complex acting as a net source or sink of DOM? By answering these questions, we provide new knowledge to better understand DOM cycling within this model treatment wetland, and the role that the wetland plays in the watershed dissolved organic carbon (DOC) budget.

## Methods

### Study site

Frank Lake (50°33′N, 113° 42′W; Fig. 1) is a restored wetland that has received treated wastewater from the Cargill meat processing plant and treated municipal sewage (town of High River) since 1989 (White and Bayley 1999, 2001; Zhu et al. 2019). Hydrologic inputs from both sources average 1,651,038 m^3^ yr^−1^ and 2,132,711 m^3^ yr^−1^ (the Town of High River and Cargill Foods Ltd), accounting for 44% and 56% of total effluent inputs, respectively. Briefly, the Cargill plant processes > 4500 cattle per day and the wastewater is treated through filtering suspended large particles, anaerobic and aerobic treatment processes, and UV disinfection before export to Frank Lake (Blue Source Canada ULC 2017). The Town of High River has a population of 14,000, and wastewater receives secondary treatment using aeration treatment processes, but without UV disinfection (www.highriver.ca). Two ephemeral creeks (Blackie and Mazeppa) discharge water to Frank Lake during the spring (Zhu et al. 2019). Blackie Creek also periodically receives untreated municipal wastewater from an upstream lagoon in the town of Blackie (population ~ 300).

Frank Lake is a multi-basin wetland complex that has four basins and is divided at the outflow of Basins 1, 2, and 3 by steel weirs (Fig. 1). Basin 1 has a surface area of 5.01 km^2^ and mean depth of 0.67 m (White and Bayley 2001; Zhu et al. 2019). Basin 2 has a surface area of 3.6 km^2^ and a similar depth to Basin 1. Basin 3 has a surface of area of 1.4 km^2^ with 0.3 m mean depth. Basin 4 is only used for back flooding from Basin 3 during wet periods (not shown in Fig. 1) (White and Bayley 1999). The wetland is fringed by emergent vegetation, primarily bulrush (*Schoenoplectus acutus* Muhl.), and contains submerged vegetation including sago pondweed (*Stuckenia pectinata*), northern water milfoil (*Myriophyllum exalbescens* Fern.) and Richardson’s pondweed (*Potamogeton richardsonii* (Benn.) Rydb.) (White and Bayley 2001).

The mean annual air temperature near Frank Lake was estimated as 2.3 °C (Zhu et al. 2019), with a monthly average mean temperature ranging from − 11 to 15 °C (White et al. 2000). From 2013 to 2015, the mean annual precipitation in the region encompassing Frank Lake was 450 mm and mean evaporation was 782.5 mm, as reported by Zhu et al. (2019) with total water losses of 332.5 mm yr^−1^. In contrast to the relatively wet 2013 to 2015 period, annual precipitation for 2021 in the region was 245 mm (Blackie AGCM; Alberta Agriculture and Forestry), which was about half the amount of precipitation received annually from 2013 to 2015, but the evaporation in 2021 was 990.4 mm (Blackie AGCM; Alberta Agriculture and Forestry), which was 1.27 times higher than in 2013–2015. Due to this extreme difference, we have categorized these distinct hydroclimatic periods as wet (2013–2015) and drought (2021) periods.

Like other aquatic ecosystems in semi-arid regions, Frank Lake experiences high rates of evaporation (7.9 × 10^6^ m^3^ yr^−1^ (Zhu et al. 2019)), which concentrates solutes as water masses move through the wetland complex. We have reported general features from previously existing data, including DOC concentrations, water temperature, and mean discharge from 2012 to 2018 (Table S1) for all sampling sites using publicly available published data from (Zhu et al. 2019) and unpublished data from Alberta Environment and Parks. Based on data from 2012 to 2018, Blackie and Mazeppa creeks had mean discharge of 0.02 to 0.03 m^3^ s^−1^ from March to June, and no flow after June. Effluent discharged year-round, with a mean of 0.12 ± 0.03 m^3^ s^−1^. Basin 3 outflow had a high flow peak in April and May and decreasing discharge rates through the year, with lowest flow in September, and a mean annual discharge rate 0.23 ± 0.32 m^3^ s^−1^.

### Water sample collection 

Routine samples were collected bi-weekly during spring and summer (March 12, 2021 to Aug 23, 2021), monthly in November and December 2020, and in September and October 2021. We sampled manually with spot-sampling at mid-day during both periods with and without flow to understand in situ DOM processing and flux, even during periods of hydrologic stagnation in the wetland and its tributaries. Surface water samples were collected from Blackie (BL) and Mazeppa (MA) Creeks, from the mouth of the pipe delivering effluent (EF) to Frank Lake, and from the outlets of Basin 1 to 3 (Fig. 1; B1O, B2O, and B3O). Given that this was a drought year with limited precipitation, water flows in creeks and at the outflow were limited. For BL, samples were collected in spring when the creek was briefly flowing (see below), and thus are limited to one data point. For MA, we observed no flow, and the samples were collected from a stagnant pool of water that remained in the creek. For B3O, samples were collected from April 9 to July 26, however flows were only observed from April 9 to May 19, with samples representing both the period of flow, and the period when flow ceased. Detailed spatial surveys of Frank Lake Basins 1 and 2 (B1, B2, respectively) were conducted by motorized boat in June and August 2021 (Fig. 1). Water for inlets and outlets was collected from shore and water from these detailed surveys was collected at a depth of ~ 0.25 m. Water was filtered through pre-rinsed 0.45 µm capsule filters (FHT-45, Waterra) or 0.45 µm filters (Cellulose nitrate membrane filter, Whatman) into acid-washed bottles or pre-combusted (450 °C, 4 h) amber glass vials within 6 h of water collection.

### Hydrologic measurements

To better constrain the period of flow, we monitored water height at each site (BL, MA, B3O) visually and using water level data loggers (HOBO® U20L; at 30 min intervals) from April 9, 2021 to September 26, 2021 (Fig. S1). Loggers were moored in the creeks with a metal stake. Water level was calculated using standard HOBO software, with reference air pressure from the nearest weather station at the CALGARY INT'L CS Station (51° 06′ N, 114° 00′ W; Government of Canada). Local precipitation and evaporation were obtained at station Blackie AGCM between November 2020 to October 2021 (Alberta Agriculture and Forestry). In all cases where discharge was observed, we attempted to measure flow using a Pygmy meter (model 625), but flow rates were below the level of detection.

### Biodegradable DOC (BDOC) experiment set up

To evaluate the capacity for the ambient microbial communities to process DOM, we conducted standardized, 28-day BDOC incubations (at room temperature, 21–23.5 °C) for water samples from all routinely monitored in- and outflowing sites in July and October. Incubations were set up in triplicate following standard protocols (Vonk et al. 2015) but with filter pore sizes changed from 0.7 µm to 0.2 µm to limit microbial activity in sub-samples from the incubation bottles. All bulk water samples were filtered to 0.2 µm (PALL Supor 200) into pre-combusted (450 °C, 4 h), 1L amber bottles and stored in the refrigerator at 4 °C for less than 24 h while setting up experiments. To begin the incubation, we added a 1% microbial inoculation (water filtered using pre-combusted 1.2 µm Whatman GF/D filters) from corresponding sites to each sample. We filtered subsamples at day 0, 2, 7, 14, 21, and 28 for DOC concentration and optical (absorbance and fluorescence spectra) measurements. We acknowledge that this closed-system approach has limited applicability to ecosystem level processes because incubations are done in the absence of environmental effects including light exposure and DOM photo-oxidation, or nutrient inputs from sources such as sediment layers or littoral vegetation. The exclusion of these processes alters microbial activity through time and can lead to underestimates of ecosystem-level rates of DOM processing under short water residence time (WRT) conditions, or overestimates under long WRT conditions (Evans et al. 2017). Despite these well-known limitations, our BDOC incubations were intended to capture relative differences in microbial DOM processing capacities between sites and dates, as a compliment to ecosystem level observations of DOM compositional change and mass balancing of DOC.

### Measurement of DOC concentrations

We determined DOC concentrations (mg L^−1^) using a Shimadzu TOC-L CPH high temperature catalytic oxidation total organic carbon analyzer calibrated with a six-point standard curve (*R*^2^ = 0.999) based on the estimated concentration range of DOC in these samples. Each sample was acidified to pH 2 (1 µl 12 N HCl per 1 ml sample water) and run on the TOC analyzer following Johnston et al. (2018). We used acidified ultrapure lab water (pH = 2) as a blank. The concentration of each sample was determined by averaging 3 of the 7 injections with the lowest coefficient of variance (C.V. < 0.02) and standard deviation (S.D. ± 0.1). The samples were run in either duplicates or triplicates and results were averaged to determine final concentrations.

### Optical properties of DOM and analysis

The measurements of absorbance (Biochrom Ultrospec 3100 pro UV–visible spectrophotometer) and fluorescence (Shimadzu RF-6000 fluorometer) were completed at room temperature using a 1 cm quartz cuvette and within 2 weeks of collection with few exceptions. Filtered water samples were generally run in triplicate. Absorbance spectra for each sample were measured from wavelengths of 230–800 nm. Blank correction was done automatically in the instrument software upon acquisition. Fluorescence spectra of each sample were measured between the wavelengths of 230–500 nm, in 5 nm intervals for excitation and 250–700 nm, in 2 nm intervals for emission. We used a scan speed of either 2000 nm s^−1^ or 6000 nm s^−1^, as determined by DOC concentration and fluorescence intensity. Fluorescence spectra were blank corrected, Raman normalized, and inner filter effect corrected in R (R Core Team 2021) using the StaRdom script (Pucher et al. 2019).

To characterize bulk optical properties of DOM in each sample, we used the StaRdom package (Pucher et al. 2019) in R to process fluorescence and absorbance data and calculate fluorescence and absorbance parameters that have been defined in previous studies (Dobbs et al. 1972; Helms et al. 2008; McKnight et al. 2001; Murphy et al. 2013). The absorption coefficient at 254 nm (*a*_254_; m^−1^) is an indicator of chromophoric DOM and specific ultraviolet absorbance at 254 nm (SUVA_254_; L mg C^−1^ m^−1^), the DOC-normalized absorbance at 254 nm, is used as an indicator of DOM aromaticity (Weishaar et al. 2003). Spectral slope ratio (*S*_R_) is the ratio of the exponential regression slope from 275 to 295 nm (*S*_275-295_) to that from 350 to 400 nm (*S*_350-400_), where *S*_R_ is inversely related to the average molecular weight of the DOM pool (Coble 2007; Cory and McKnight 2005; Helms et al. 2008; Spencer et al. 2012). Similar to SUVA_254_, fluorescence index (FI) is an indicator of DOM aromaticity but has an inverse relationship to aromaticity (Cory and McKnight 2005; Findlay and Sinsabaugh 2003).

We calculated the widely reported fluorescence peaks using the excitation emission matrices for each sample, B (ex/em = 270 nm/310 nm), T (ex/em = 275 nm/340 nm), A (ex/em = 260 nm/380 to 410 nm), M (ex/em = 312 nm/380 to 420 nm), and C (ex/em = 350 nm/420 to 480 nm), which generally correspond to protein-like (B and T, more bio-available) and humic-like (A, C, and M, less bio-available) DOM availability (Fellman et al. 2010). We also calculated the A:T peak ratio, which is an indicator of the relative amount of less bio-available to more bio-available fluorescent DOM in a sample.

Fluorescence data from routine samples were analyzed using parallel factor analysis (PARAFAC) (Murphy et al. 2010; Pucher et al. 2019; Stedmon & Bro 2008) in R (R Core Team 2021) to determine the best fit number of component model and to compare with previously published results in the OpenFluor database (Murphy et al. 2014). After fluorescence spectra were corrected, a total of 50 samples were used for PARAFAC model development, and 5 outliers were removed for validation of the model using split-half analysis (> 95%). The five outliers were EF on July 13 and 26 and Aug 23, B2O on July 26, and BL on May 10.

### Mass balance construction and relation to WRT

We built a DOC mass balance for Frank Lake following the general methods of Evans et al. (2017), and compared the wet period (2013–2015) to the drought period during which we collected our samples (2021), then combined both to determine the long-term budget. To construct the mass balance, we estimated the annual mass of DOC entering and leaving Frank Lake (Mg C yr^−1^):

1 $${\text{Net DOC flux }} = {\text{ DOC}}_{{{\text{mass - out}}}} - \Sigma {\text{DOC}}_{{\text{mass - in}}}$$

where the DOC input (Ʃ DOC_mass-in_) was the sum of inputs (EF, BL, MA). Output (DOC_mass-out_) included B3O (Fig. 1). Positive flux indicates that Frank Lake is a net DOC source (enhancing output of DOC downstream), and a negative flux represents a net DOC sink (reducing outputs of DOC downstream).

The individual masses of DOC outputs or inputs at each site were calculated as the sum of bi-weekly to monthly estimates of the product of water flux (*Q*_month_) and averaged DOC concentrations (DOC_conc_):

2 $${\text{DOC}}_{{\text{mass - in}}} {\text{ or DOC}}_{{\text{mass - out}}} = \Sigma \left( {{\text{Q}}_{{{\text{month}}}} {\times{\text{DOC}}}_{{{\text{conc}}}} } \right)$$

DOC concentrations from 2013 to 2015 were obtained from Alberta Environment and Parks, and for 2021, from our own measurements.

For *Q*_month_ in the drought period of 2021, monthly effluent release volumes (Nov. 2020–Oct. 2021) were obtained from the town of High River and Cargill Foods Ltd. In 2021, we observed no discharge in MA. For BL, we observed a brief period of discharge (~ 5 days) associated with the release from the upstream Blackie lagoon (total discharge volume of 15,000 m^3^; data from Foothills County, Alberta). This total volume was averaged to determine daily discharge in May 2021. For B3O, flows were below detection limits for our flow meter, so we used the estimated daily average discharge values (*Q*; m^3^ s^−1^) from the Government of Alberta Flow Estimation Tool for Ungauged Watersheds (AFETUW) (https://afetuw.alberta.ca/). These mean daily estimates from the model were constrained to the period of observed flow based on visual observations and estimates of water height (from the logger). As our goal was to use discharge data to construct a first order budget of DOC flux for the wetland, these estimates of discharge are more than sufficient, because all estimates of inflow (< 1%) and outflow (< 18%) presented here for 2021 were minor relative to annual discharge from EF, and small errors associated with these flux calculations are minor relative to the comparatively well-constrained estimates of effluent discharge and DOC concentrations.

For the wet period of 2013–2015, mean annual EF discharge values were obtained from the Town of High River and Cargill Foods Ltd. and DOC data were converted to an annual average (Table S1). Discharge rates for all other sites were directly measured by Zhu et al. (2019) using the velocity-area calculation approach with a handheld velocity meter. We used these published data to calculate *Q*_month_ since estimates of *Q*_yr_ from the AFETUW model for 2013–2015 for BL and MA were 40 to 60% higher than direct estimates, beyond reasonable expectations for flow in the creeks. They also included periods of flow when none were observed visually (Zhu et al. 2019). Mean daily *Q* (m^3^ s^−1^) observations (Table S1) were averaged into monthly mean values when flow was observed (*Q*_month_). The same monthly averaging was applied to DOC concentration data (Table S1). To overcome gaps in our dataset, for the 2013–2015 wet period, we averaged *Q*_month_ and DOC concentrations across all 3 years for each month. For the overall (all years) average DOC flux values, we used the same method as for the 2013–2015 period but included data from 2021. Annual discharge (*Q*_yr_, m^3^ yr^−1^) and annual DOC fluxes (Mg yr^−1^) were determined by summing monthly mean values.

We determined annual WRT for Frank Lake using the mean total volume of the wetland complex from Zhu et al. (2019) divided by the mean *Q*_yr_ from Basin 3 outlet (calculated for the combined 2013–2015 wet period, for the 2021 drought period, and as a long-term average using all four years). To remain consistent with past work (Evans et al. 2017), we do not consider evaporation in calculations of WRT, and therefore only present the ratio of lateral exchanges of the mass of DOC. Based on the conclusions of Zhu et al. (2019), we assumed that groundwater influence on the DOC mass balance was negligible. These authors concluded that relative to other flows, groundwater contributed ~ 4% to the total water inputs in Frank Lake, and further supported these findings with a salt budget and water isotope results. If we extend these past estimates to the more recent drought period when our samples were collected (see results below), the potential input of groundwater (0.49 ± 0.05 × 10^6^ m^3^ yr^−1^) identified by Zhu et al. (2019) would represent ~ 14% of 2020–21 input flows, but given the order of magnitude lower DOC concentration in regional groundwater (Wassenaar et al. 1991) than in Frank Lake effluent, the importance of groundwater as a DOC source in our budget would be negligible during the drought period. A strong linear relationship between DOC concentrations and salinity through the basins in Frank Lake in 2021 (Bogard et al. under review) further indicates that a dilution effect of low-DOC groundwater on the Frank Lake surface DOC pool is absent.

### Modelling DOC consumption in BDOC incubations

We modelled the changes in DOC content during the BDOC experiment and reactivity of distinct DOM pools following existing methods of Catalán et al. (2016) and Guillemette and del Giorgio (2011). The decay rate *k* for each site was calculated as *k* = ln(*t*/*i*)/*T*, where the rate of decomposition is defined as ln (*t*/i), with *t* indicating DOC concentration at time *t* (on day 28 of incubations), *i* indicating initial DOC concentration (on day 0), and *T* the duration of the incubation. We estimated the half-life of the DOC pool as: *t*_1/2_ = ln(2)/*k*.

### Statistical analyses

All analyses were performed in R version 4. 1. 2 (R Core Team 2021). We used analysis of variance (ANOVA) with Tukey’s honestly significant difference post hoc tests (Kao & Green 2008) (*Tukey_hsd* function) to compare DOC concentrations, BDOC concentrations, and optical parameters across sites except for Blackie and Mazeppa Creeks. Data were transformed to meet assumptions of normality only where data violated these assumptions as identified using the Shapiro-Wilks test (*Shapiro* function). If a dataset failed the test of normality, a non-parametric analysis (Kruskall-Wallis) (*Kruskal.test*) with Wilcoxon signed-rank test (*Wilcox.text*) was used to evaluate inter-group differences. We used the *lm* function for all linear regression calculations.

## Results

### General properties of the Frank Lake wetland complex

In 2021, the brief period of wastewater discharge from the Town of Blackie lagoon caused a ~ 5-day period of flow at BL in May (Fig. S1). B3O had a consistent water height from April 9 to May 29 and we did not observe flow beyond May 29 with water height consistently decreasing after that time (Fig. S1). Estimated discharge for B3O from the AFETUW model was 0.116 ± 0.05 m^3^ s^−1^ (mean ± S.D.) for the flow period. During the sampling period, daily precipitation was low (Fig. S1) with a mean daily value of 1 ± 4 mm (April 9 to September 22), and maximum of 43 mm (August 17).

### DOC concentration and DOM composition

**Fig. 2:**
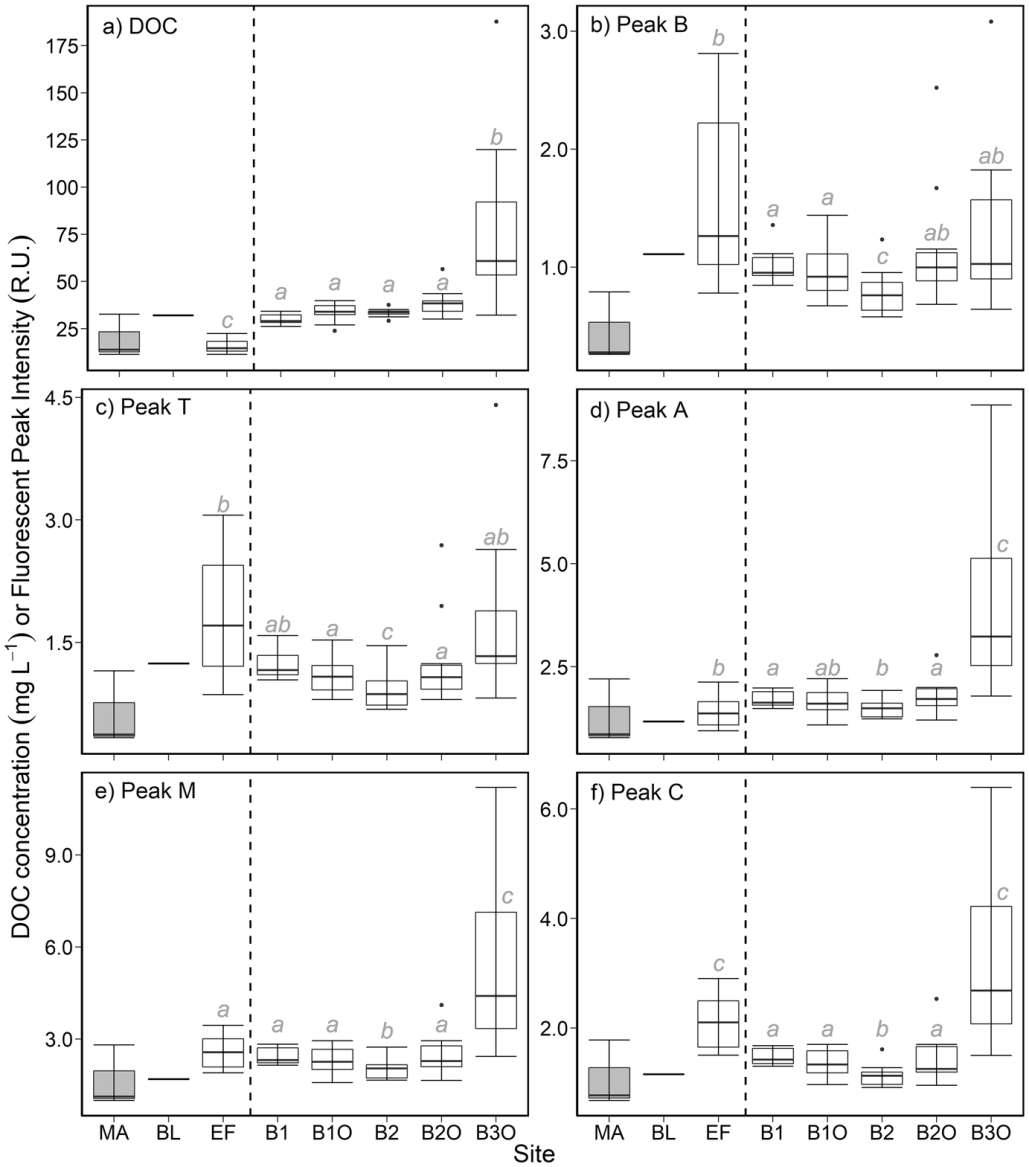
Differences in DOC concentration (mg L^−1^; panel **a**) and fluorescent DOM (FDOM) composition (R.U.; B, T, A, M, and C peaks; panels **b**–**f**) among sites at the Frank Lake wetland complex. The dashed lines separate inlet sites, MA, BL, and EF (left), from basin outflows (B1O, B2O, B3O) and spatial surveys (B1 and B2) (right). All ANOVA (DOC) and Kruskal–Wallis (B, T, A, M, and C peaks) results showed significant group differences (*p* < 0.001) with statistically significant post hoc comparisons (*p* < 0.05) denoted by grey, italicized, lower case letters. BL and MA (grey boxes) were not included in statistical comparisons as only one data point was available for BL, and water from MA was stagnant and not contributing to Frank Lake

The concentration of DOC varied among sample sites (Fig. 2a; ANOVA: *p* < 0.01, *n* = 78) more widely than within sites, and EF and B3O had significantly different DOC concentrations than other sites (lower, and higher, respectively). MA had intermediate concentrations compared to both sites (15.5 ± 3.3, 32.0, and 19.4 ± 11.6 mg L^−1^ respectively for EF, BL, and MA). Concentrations of DOC increased from 33.7 ± 4.7 mg L^−1^ at B1O, to 38.4 ± 7.1 mg L^−1^ at B2O, to 81.0 ± 47.4 mg L^−1^ at B3O. The DOC concentration ranges in spatial surveys of B1 and B2 were not statistically different from those at the seasonally sampled outflow locations (Fig. 2a). Absorption coefficients (*a*_254_; m^−1^) followed generally consistent patterns with DOC concentrations (Fig. S2).

Overall, the DOM pool at EF appeared to be unique, with more bio-labile fluorescent DOM (FDOM) relative to all other sites (Fig. 2b–f). Across the basins, fluorescence peak intensities showed the same pattern as for DOC concentrations, increasing from B1 to B2O, with a dramatic increase at B3O (Fig. 2b–f). Fluorescence peaks A, M, and C (Kruskal–Wallis: *p* < 0.001, *n* = 78) were consistently more intense at B3O than other sites, and lower at B2 than other sites (except for peak A). B and T peak intensity at EF was greater than B1O and moderately greater than at other sites (Kruskal–Wallis: *p* < 0.001, *n* = 78), while B3O was significantly different from B2. Generally, the FDOM peak intensities were higher in EF and B3O sites and had no clear shift from inflow to outflow locations.

**Fig. 3:**
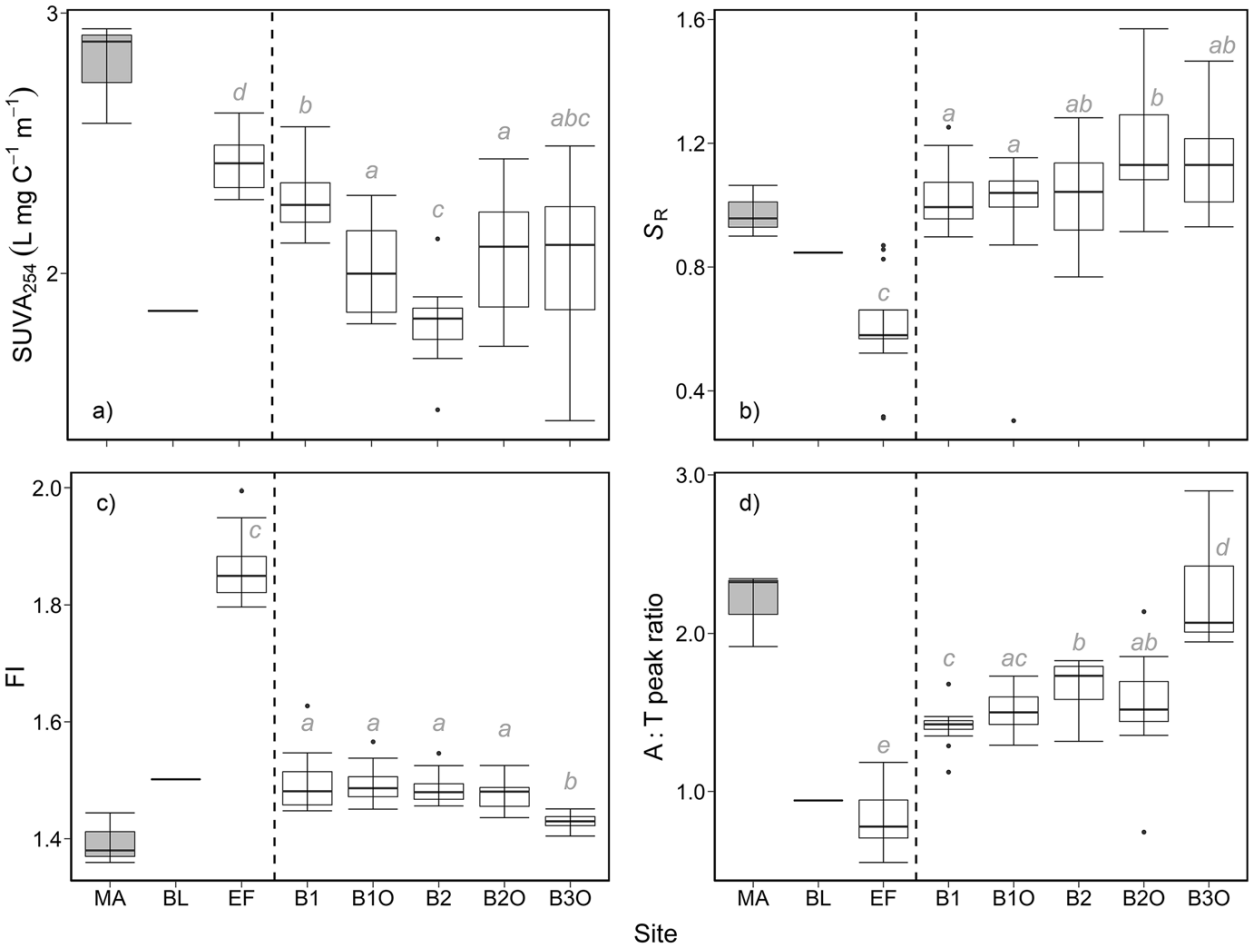
Compositional differences in DOM across sites. Panels show the specific UV absorbance at 254 nm (SUVA_254_; panel **a**), spectral slope ratio (*S*_R_; panel **b**), fluorescence index (FI; panel **c**) and the Peak A to T intensity ratio (A:T; panel **d**). Formatting and statistical presentations as in Fig. 2. Differences among groups were observed for all metrics (Kruskal–Wallis test; *p* < 0.001; *n* = 78)

Optical properties indicated that the relative composition of DOM at the EF site was distinct from other sites (Fig. 3). SUVA_254_ values at EF (2.4 ± 0.1 L mg C^−1^ m^−1^) were comparable to those for MA (2.8 ± 0.2 L mg C^−1^ m^−1^; Fig. 3a), and greater than values from the downstream Basins (Kruskal–Wallis, *p* < 0.001, *n* = 78). SUVA_254_ values decreased by B2 (to 1.8 ± 0.1 mg C^−1^ m^−1^) then increased at B3O (to 2.0 ± 0.3 mg C^−1^ m^−1^). Values of *S*_R_ at EF (0.60 ± 0.17) were lowest (Kruskal–Wallis, *p* < 0.001, *n* = 78) followed by the other inlets BL (0.85) and MA (0.97 ± 0.08) (Fig. 3b). *S*_R_ values were relatively consistent from B1 to B2 (~ 1.0). Across sites, FI values showed similar but opposite patterns compared to *S*_R_ (Fig. 3c). The EF site had the highest FI values (1.86 ± 0.05), and MA and BL had values of 1.39 ± 0.04 and 1.50, respectively. Among the basins, FI values were relatively consistent from B1 to B2O (~ 1.48) then decreased to 1.43 ± 0.01 at B3O (Kruskal–Wallis, *p* < 0.001, *n* = 78; Fig. 3c). The A:T ratio showed a similar pattern to *S*_R_ values with the lowest ratio observed from samples of EF (0.83 ± 0.19) (Fig. 3d). Ratios of A:T increased from B1 (1.41 ± 0.12) through B2O (1.54 ± 0.33) and B3O (2.25 ± 0.36). Only EF and B3O had A:T ratios that were statistically different from all other sites (Kruskal–Wallis, *p* < 0.001, *n* = 78; Fig. 3d).

### PARAFAC model results

**Fig. 4:**
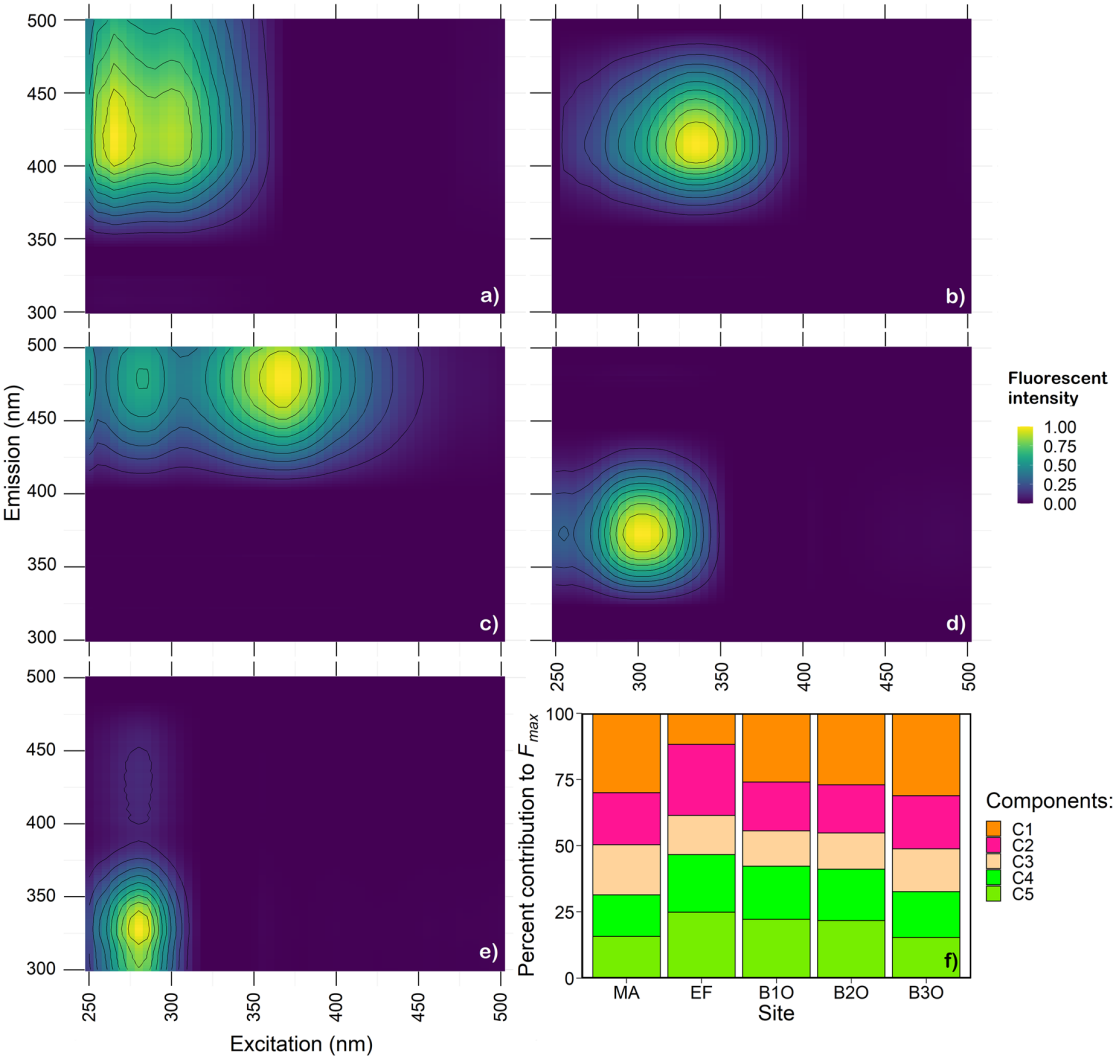
The percent contribution the of five fluorescent components (C1–C5; panel **a**–**e**; respectively) assigned using PARAFAC at each routine sampling site (panel **f**). Panels **a**–**e** are exitation emission matrices for C1–C5, respectively. Each component in panel **f** is scaled to *F*_*max*_, and components for each site sum to 100%

**Fig. 5:**
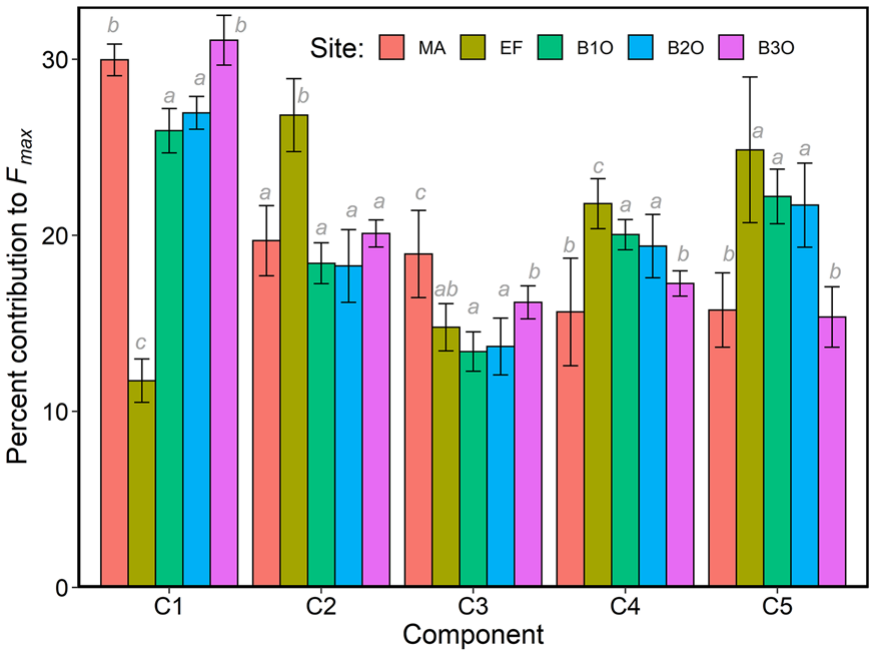
Percent contribution to *F*_*max*_ for C1 (Ex/Em = 265/422 nm), C2 (Ex/Em = 235/416 nm), C3 (Ex/Em = 265/478 nm), C4 (Ex/Em = 305/372 nm) and C5 (Ex/Em = 280/328 nm). All sites are shown except BL, which was excluded to generate a significant PARAFAC model. Significant differences (ANOVA) are summarized with grey italicized letters that denote inter-group differences as defined by Tukey HSD post hoc comparisons (*p* < 0.001, *n* = 45). Error bars represent ± 1 S.D

Our PARAFAC model contained five components (C1–C5; Fig. 4). DOM from EF showed a unique sequence of components compared to other sites (Fig. 5; Table S2). Component C1 had the highest percent contribution at all sites except EF (ANOVA, *p* < 0.001, *n* = 45), and represents humic-like DOM common in terrestrial environments (Coble 2007; Wünsch et al. 2017). The percent contribution of C1 increased from EF to B3O and showed no significant difference between B1O and B2O. Unlike other sites, EF had DOM with the highest percent contribution of C2 (ANOVA, *p* < 0.001, *n* = 45), a component that has previously been related to wastewater or nutrient-rich surface waters (Jutaporn et al. 2020; Murphy et al. 2011). For C2 and C3, we saw a decrease in percent contribution moving through the wetland complex from EF to B2O, then an increase from B2O to B3O. C3 is a mix of peak A and C (Yamashita et al. 2011). MA had a high percent contribution of C3 compared to other sites (ANOVA, *p* < 0.001, *n* = 45). Percent contributions of both C4 (microbial humic-like DOM (DeFrancesco & Guéguen 2021)) and C5 (tryptophan-like DOM (Coble 2007)) decreased from EF to B3O and were lowest at MA and B3O (ANOVA, *p* < 0.001, *n* = 45). The percent contribution to *F*_*max*_ (Fig. 4) for each component ranked differently for EF (C2 > C5 > C4 > C3 > C1) than B1O and B2O (C1 > C5 > C4 > C2 > C3; Fig. 4), and B3O and MA, which had the same order for the first three components (C1 > C2 > C3).

### Biodegradable DOC (BDOC) incubations

**Fig. 6:**
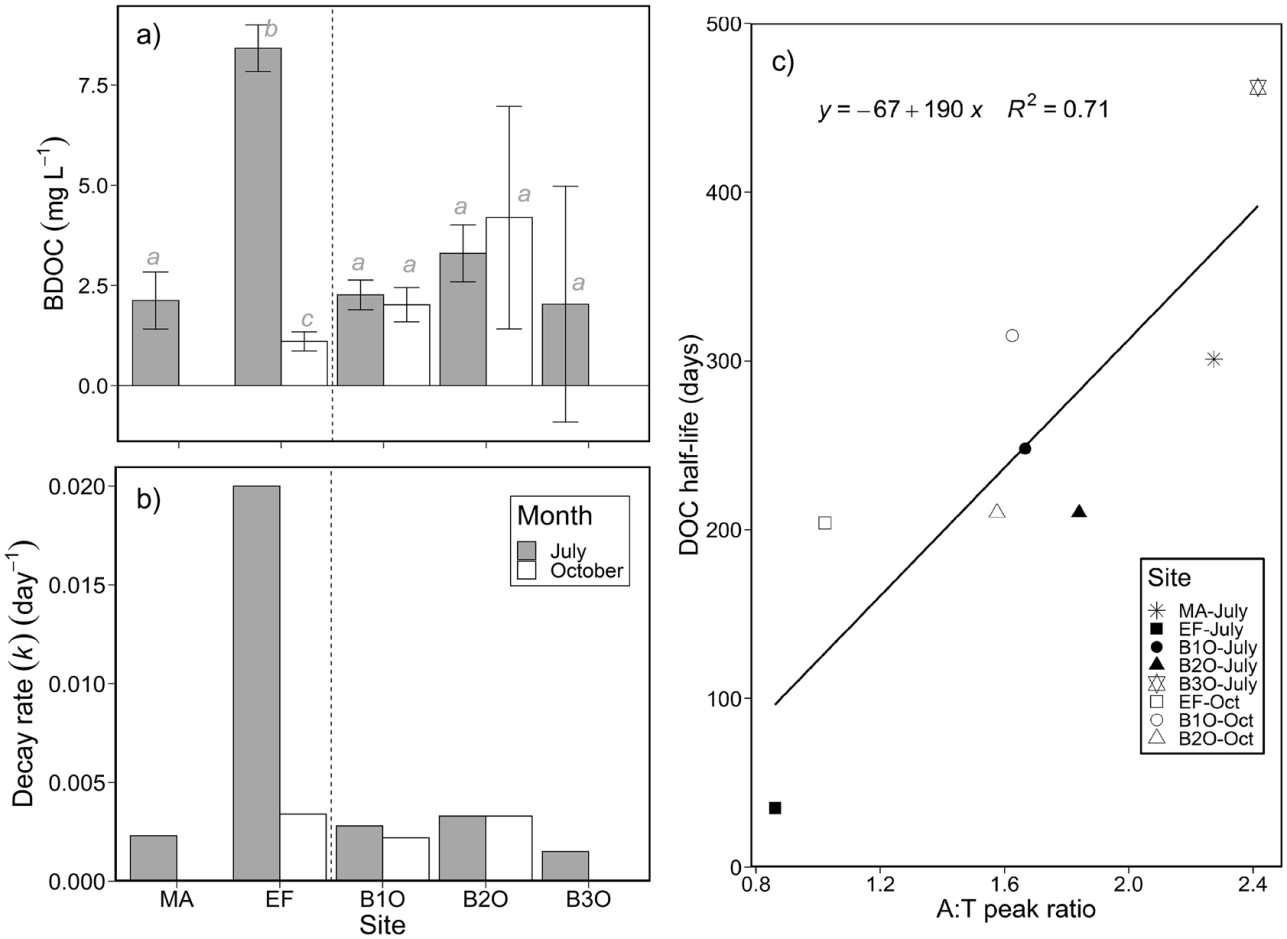
Microbial incubations showed distinct DOC processing through the wetland and through time. Biodegradable DOC values (BDOC; day 0 minus day 28 DOC concentrations; panel **a**) with clear inter-group differences (ANOVA, *p* < 0.001, *n* = 30; error bars represent ± 1 S.D). DOC decay rates (panel **b**) from DOC concentration change (Fig. S4) for each site and sample period. The relationship between the A:T fluorescence peak ratio at the onset of incubations, and the half-life (*t*_1/2_) of the DOC pool (panel **c**) from July to October

Concentrations of DOC and DOM optical properties at the onset of all BDOC experiments (Table S3) were consistent with values observed in ambient water samples (Figs. 2 and 3). Total DOC consumption during incubations was greatest for the EF site in July (8.4 ± 0.58 mg L^−1^), while no difference was found across the rest of the sites that all had lower total DOC removal in July (~ 2.0–3.3 mg L^−1^) and in October (~ 1.1–4.2 mg L^−1^) (ANOVA: *p* < 0.001, *n* = 30; Fig. 6a). Based on BDOC consumption over 28 days for each site in July (Fig. S3; Table S3), EF had the highest decay rate, *k* (0.02 day^−1^), greater than for MA (0.0023 day^−1^), B1O and B2O (~ 0.003 day^−1^), and B3O (0.0015 day^−1^) (Table S3; Fig. 6b). In October, the BDOC incubation for EF had a similar *k* value as B2O (0.0034 and 0.0033 day^−1^, respectively). Both EF and B1O (but not B2O) showed a decrease of *k* from July to October. The half-life of DOC in July for these incubations (*t*_1/2_; Fig. 6c, Table S3) was lowest at EF (35 days) and highest at B3O (462 days). Both B1O (248 days) and B2O (210 days) had intermediate *t*_1/2_ values. In October, the *t*_1/2_ for EF increased to 204 days, and B1O increased to 315 days, while B2O was unchanged. The patterns of DOC removal in the incubations were strongly linked to the composition of DOM, and the ratio of A:T fluorescence peaks were strongly positively correlated with *t*_1/2_ (*R*^2^ = 0.71; *p* = 0.009; Fig. 6c).

Over the course of BDOC incubations, we observed large changes in DOM composition for EF, but not in water from the other sites. From day 0 to 28 in the July EF incubation, we saw a clear (~ 0.09 units) shift in FI values toward more aromatic DOM composition, and large but varied decreases in all individual fluorescence peak intensities (Fig. S4). Among the incubations from the other sites in July, we observed consistent decreases in the intensity of B and T peaks, little change for A peaks, and increased intensity in M and C peaks. In the October incubations, we observed smaller changes in peak intensities that were generally in the same direction as those in July, except for EF, B1O and B2O, for which the direction of change for peaks B, T, and A were opposite to those in July (Fig. S4).

### Mass balance of lateral DOC flux across distinct hydrologic conditions

The averaged lateral water flux (defined as *Q*_yr_ of output at B3O minus the sum of *Q*_yr_ of inputs from BL, MA, and EF sites) at Frank Lake was − 1.44 ± 0.61 × 10^6^ m^3^ yr^−1^. Water added to Frank Lake from the EF inlet accounted for 82 to 99.5% of total hydrologic inputs to Frank Lake among all years (3.82 ± 0.31 × 10^6^ m^3^ yr^−1^; Table 1). During the wet period (2013–2015), BL and MA imported 0.43–0.45 × 10^6^ m^3^ yr^−1^ water while B3O exported 3.91 ± 0.60 × 10^6^ m^3^ yr^−1^. The net water flux during this time at Frank Lake was − 0.92 ± 0.74 × 10^6^ m^3^ yr^−1^. During the drought period of 2021, BL imported 0.015 × 10^6^ m^3^ yr^−1^ 2021 (Table 1) and MA did not flow. The net water flux in 2021 was − 2.88 ± 0.61 × 10^6^ m^3^ yr^−1^. The negative net water flux in both wet and drought periods represent water loss from evaporation, assuming no groundwater recharge (Zhu et al. 2019; see methods for more details about this assumption). WRT was 3.27 years averaged across all four years but increased from the 2013 to 2015 wet period (2.56 years) to the 2021 drought period (16.4 years).

**Table 1:** Mean annual discharge and DOC flux for wet and drought periods, and all years averaged. Standard deviation in parentheses if applicable

Hydrologic phase (year(s))	Site	Discharge (10^6^ m^3^ yr^−1^)	DOC (mg L^−1^)	DOC flux (Mg C yr^−1^)	Net DOC flux (Mg C yr^−1^)
Wet period (2013 to 2015)	BL	0.43 (0.07)	24.4 (4.8)	9.5 (2.0)	
	MA	0.45 (0.26)	21.9 (6.0)	9.4 (5.7)	
	EF	3.94 (0.30)	11.7 (3.1)	45.9 (12.7)	
	B3O	3.91 (0.60)	31.6 (8.4)	117.9 (22.9)	
	Total				53.0 (26.9)
Drought period (2021)	BL	0.015	32.03	0.48	
	EF	3.47	15.5 (3.3)	53.7 (11.3)	
	B3O	0.61 (0.24)	49.4 (12.2)	29.8 (13.5)	
	Total				− 24.4 (17.6)
All years	BL	0.33 (0.05)	25.4 (5.2)	7.3 (1.5)	
	MA	0.34 (0.02)	21.9 (6.0)	7.2 (4.3)	
	EF	3.82 (0.31)	12.8 (3.6)	48.9 (14.3)	
	B3O	3.06 (0.50)	33.2 (10.2)	103.1 (19.7)	
	Total				39.7 (24.7)

The import of DOC to Frank Lake from EF varied little among years but ranged in relative importance from 71% (45.9 ± 12.7 Mg C yr^−1^) of total inputs in the wet period to 99% (53.7 ± 11.3 Mg C yr^−1^) in the drought period (Table 1). This variability was largely due to differences in water flux since long term averages of DOC concentrations were comparatively stable at EF, BL, and MA (11.7 ± 3.1, 25.4 ± 5.2, and 21.9 ± 6.0 mg L^−1^, respectively). Long term average inputs from BL and MA were small (7.3 ± 1.5 and 7.2 ± 4.3 Mg C yr^−1^, respectively; Table 1), but ranged widely among years. During the wet period MA and BL contributed 9.4 ± 5.7 and 9.5 ± 2.0 Mg C yr^−1^, respectively, while fluxes were extremely small in the drought period (MA was zero, and BL imported 0.48 Mg C yr^−1^). The output at B3O averaged 103.1 ± 19.7 Mg C yr^−1^ across all years, ranging from 29.8 ± 13.5 Mg C yr^−1^ (drought period) to 117.9 ± 22.9 (wet period). Frank Lake was a net DOC source over all years (39.7 ± 24.7 Mg C yr^−1^; Table 1), but net DOC flux differed dramatically from wet (53.0 ± 26.9 Mg C yr^−1^) to drought periods (− 24.4 ± 17.6 Mg C yr^−1^). The mean ratio of DOC output at B3O to total DOC inputs (DOC_OUT_/DOC_IN_) was 1.63 across all years (1.82 and 0.55 during the wet and drought period, respectively).

## Discussion

Wetland processing of effluent DOM is an important service to society. Yet net outcomes of DOM cycling can vary widely from one wetland to the next due to a multitude of controls that include but are not limited to the chemical composition of effluent DOM, hydroclimatic conditions, and internal processing and turnover of the DOM pool (Barber et al. 2001; Pinney et al. 2000). We show that the Frank Lake wetland complex effectively mineralizes and modifies effluent DOM, despite being a long-term (multi-year) net source of DOM to downstream environments (Table 1). Within the wetland complex, DOC concentrations increased, and the DOM composition shifted from more protein-like at EF, towards more humic-like at the outlet of the wetland at B3O, which provides a clear indication of active DOM processing in transit (Fig. 2). Consistent with this interpretation of compositional shifts through the wetland, our incubations showed that DOM is actively mineralized by wetland microbes. The half-life of the DOC pool in our incubations was shortest in water masses entering the wetland but increased through the basins, tracking the shift toward more humic-like and less bioavailable DOM (Fig. 6). This shift in DOM composition is consistent with some, but not all treatment wetlands (Barber et al. 2001; Clark et al. 2020). Despite being an overall net source of DOM over a longer, multi-year timescale, the source or sink strength of Frank Lake shifted between wet and drought periods that this region is prone to (DOM source versus sink, respectively, Table 1, Fig. S5). This indicates that the role of the wetland complex in the regional aquatic network shifts over interannual timescales depending on climatic and thus hydrometeorological conditions, and emphasizes the need to consider single-year studies of wetland DOM processing with caution when the goal is to evaluate the source or sink strength of a given ecosystem. Together, our findings help characterize the capacity for mineral wetlands like Frank Lake to act as systems for organic effluent processing. This work is especially unique given that Frank Lake is a restored, natural wetland, and most work to date has focused on DOM processing in constructed wetlands.

### Frank Lake wetland is an overall source of DOC

**Fig. 7:**
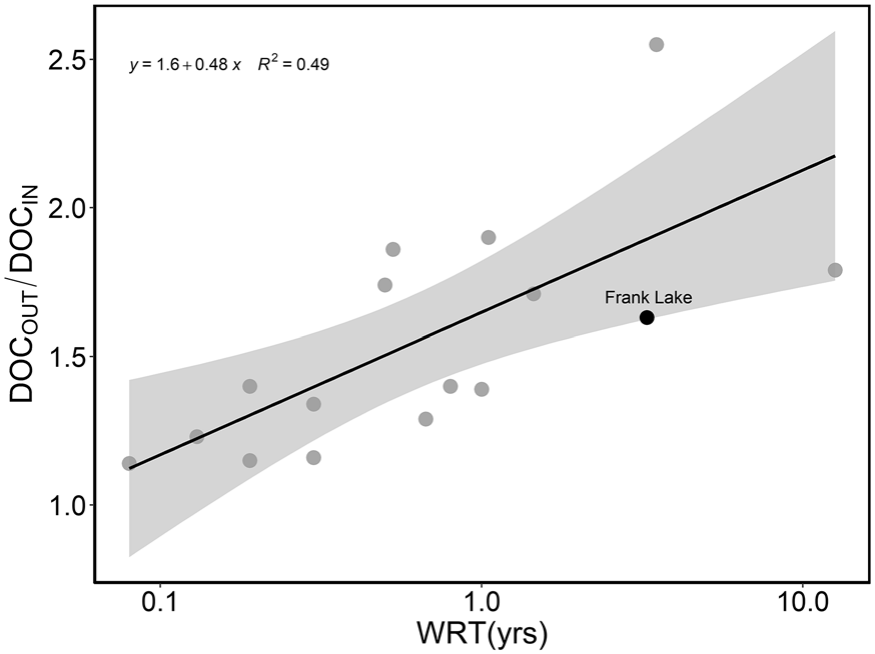
DOC export ratio of Frank Lake (FL) relative to other lentic systems that are net exporters of DOC. The long-term average ratio of DOC mass output to input (DOC_OUT_/DOC_IN_; from Table 1) and water residence time (WRT) for FL was compared to lentic system data from Evans et al. (2017), including their reported linear regression relationship (black line with the 95% confidence interval in grey)

Frank Lake is a net source of DOC with a long-term ratio of export to import (DOC_OUT_/DOC_IN_) of 1.63. This is consistent with empirical predictions based on WRT (Evans et al. 2017) (Fig. 7), suggesting Frank Lake functions similar to other inland water systems, in terms of DOM processing. Evans et al. (2017) showed a positive relationship between log (WRT) and the ratio of DOC_OUT_/DOC_IN_ for lentic systems that act as net sources of DOC. There, the ratio of DOC_OUT_/DOC_IN_ was not clearly controlled by the nutrient status of inland water systems, but by WRT. Therefore, while the extreme nutrient content (White et al. 2000; White and Bayley 2001; Zhu et al. 2019) likely enhances autotrophic production and DOM transformation in Frank Lake, this had less impact on the net balance of DOC processing. Although Frank Lake exports a large amount of DOC annually (103.1 ± 19.7 Mg C yr^−1^), this is equivalent to 10.3 ± 2.0 g C m^−2^ yr^−1^ when scaled to the wetland surface area (10.1 km^2^, Zhu et al. 2019), and is consistent with other natural wetland systems including a brackish tidal marsh (9.7 ± 2.2 g C m^−2^ yr^−1^) (Bogard et al. 2020), mangrove systems (12 g C m^−2^ yr^−1^) (Dittmar et al. 2006), and low relative to temperate wetlands (36 ± 12 g C m^−2^ yr^−1^) (Clair et al. 2002). To our knowledge, few mass balance estimates including both in- and outflow exchange rates exist for DOC flux in natural or constructed treatment wetlands, because most studies are restricted to comparisons of concentration differences of DOC (e.g., Barber et al. 2001; Clark et al. 2020; Pinney et al. 2000) or differences in optical properties (e.g., Clark et al. 2020; Yao et al. 2016) between wetland in- and outflows. Thus, by comparing Frank Lake to other inland water systems (Fig. 7) and linking the export rate to hydrologic residence time, we provide empirical evidence that compliments experimental work (Pinney et al. 2000; Vähätalo and Wetzel 2008) to ultimately explain how treatment wetlands process DOM.

Frank Lake switched from being a net DOC source to sink between wet and drought periods, respectively (Table 1, Fig. S5). This difference in DOC cycling is linked to hydrological changes that shifted the WRT from 2.56 to 16.40 years (Fig. S5). The shorter WRT in the wet period can limit DOM exposure to mineralization and photodegradation processes (Granéli et al. 1996; Vachon et al. 2021). During the drought period, the longer WRT increased the potential for in situ DOM removal by multiple mechanisms (e.g., respiration, photodegradation, burial, or assimilation). It is likely that photodegradation would preferentially remove aromatic DOM (Clark et al. 2008; Vähätalo and Wetzel 2008; Waiser and Robarts 2004) while biodegradation would tend to mineralize protein-like or LMW DOM (Findlay and Sinsabaugh 2003; Hutchins et al. 2017). More work is needed to identify the exact mechanisms driving the DOM sink in Frank Lake. However, our findings demonstrate that caution is needed when attempting to attribute a net DOM sink or source status to effluent treatment wetlands (Barber et al. 2001), particularly when datasets do not cover the range of representative interannual hydrologic conditions that a given wetland may experience.

### Frank Lake efficiently processes effluent-derived DOM

The half-life of the DOC pool at each site (35–462 days; Fig. 6) is shorter than overall WRT (mean of 3.27 years; Fig. 7), suggesting Frank Lake mineralizes most effluent-derived DOM. This conclusion is consistent with observations from other diverse treatment wetlands (Barber et al. 2001; Li et al. 2008; Pinney et al. 2000). Our observed DOC decay coefficients (*k*) decreased from 0.02 day^−1^ to 0.0015 day^−1^ from the EF inlet site to B3O outflow (Fig. 6b). These are within the range of values from comparable incubations that spanned from 0.0066 ± 0.0109 day^−1^ in small wetlands with 0.06 ± 0.25 years WRT (Catalán et al. 2016), to 0.0293 day^−1^ in small marsh wetlands with WRTs of 0.77 years (Guillemette & del Giorgio 2011). The rates of microbial DOC processing varied with DOM composition (as A:T peak ratios; Fig. 6c), reflecting the role that DOM availability plays in controlling metabolism of the microbial community (Li et al. 2008; Logue et al. 2016). Seasonal BDOC differences were minor for all but the EF for July, and BDOC processing decreased with the downstream shift toward more humic-like DOM and relatively less protein-like DOM. This trend is consistent with expectations for longitudinal DOM processing patterns in another wetland system (Pinney et al. 2000). While laboratory incubations do not encompass all ecosystem-level DOM processing, BDOC incubations do capture general DOM cycling patterns in aquatic systems (Kelso et al. 2020), so we are confident that the high mineralization rate of effluent DOM observed in incubations extends to the ecosystem scale, especially since this conclusion is supported by the 2021 mass balance that showed intense net DOM consumption.

### Compositional shifts in DOM along the hydrological continuum

As in other treatment wetlands (Barber et al. 2001), we observed a compositional shift in the DOM pool from the effluent-derived signature that is more bioavailable but higher in molecular weight, toward more aromatic, humic-like wetland-derived DOM that reflected mixed potential inputs that may diversify the composition of the DOM pool. Among sites, effluent derived DOM had the lowest DOC concentration (~ 15.5 mg L^−1^), highest FI values (~ 1.86), relatively high SUVA_254_ (~ 2.4 L mg C^−1^ m^−1^), and higher molecular weight based on *S*_R_ (~ 0.6; Figs. 2 and 3). This is comparable to previous work showing treated municipal wastewater also had SUVA_254_ values around 2.2 L mg C^−1^ m^−1^, with higher molecular weight DOM after secondary treatment, indicating the presence of microbially-derived DOM, proteins and polysaccharides (Maizel and Remucal 2017). However, these values depend on effluent sources and treatment processes, such that effluent SUVA_254_ values have been shown to vary from 0.7 to 2.9 L mg C^−1^ m^−1^ (Wang & Chen 2018), with the EF site at Frank Lake on the upper end of this reported range. Consistent with SUVA_254_ values that suggest an abundance of more aromatic DOM in effluent, PARAFAC results showed effluent also had a large relative contribution of C2, indicating an abundance of humic-like DOM, consistent with wastewater and other nutrient rich environments (Jutaporn et al. 2020; Murphy et al. 2011). DOM from the EF site also had a relatively large contribution of microbial humic-like DOM (C4) (DeFrancesco and Guéguen 2021) and tryptophan-like DOM (C5) (Osburn et al. 2011), similar to municipal and domestic sewage from other studies, possibly due to leaching of DOM from microbes during biological treatment in secondary wastewater processing (Wang and Chen 2018). Effluent DOM was likely replaced via DOM leaching from riparian and emergent vegetation and wetland soils (Clark et al. 2008; Pinney et al. 2000), especially below the outflow of Basin 2. PARAFAC component C2 decreased, while C1 (humic-like terrestrial DOM; Wünsch et al. 2017) and C3 increased significantly at B3O (Fig. 5). Further, FI values decreased to those characteristic of other natural wetlands (1.30 to 1.58; Hertkorn et al. 2016; Lu et al. 2003)). The decrease of C4 and C5 across Basins are consistent with photodegradation and biodegradation processes removing significant portions of this DOM pool (discussed above). Taken together, Frank Lake not only removes effluent DOM (Fig. 6), but it modifies the composition of the DOM pool (Figs. 3 and 5), and this study helps to pinpoint the zone of most intense DOM modification (below Basin 2). This zone is more terrestrial-like and shallow (see methods and Fig. 1), so water likely interacts with soils and emergent vegetation more effectively, leading to the observed compositional shifts in DOM. Further work is needed to determine whether this compositional shift has water quality and toxicological implications.

## Conclusions

Here, we provide new information regarding the role of an economically-important, model treatment wetland that receives multiple sources of complex effluent. Consistent with numerous other wetlands and inland water, Frank Lake appears to be a net source of DOM to downstream ecosystems. While effluent is efficiently mineralized, a large fraction appears to be replaced with internally-derived DOM, thereby shifting DOM quantity and quality toward properties of DOM reflecting wetland sources, prior to export. Further, by quantifying net DOC flux individually between wet and drought periods, we show that treatment wetlands can switch from net sources to sinks of DOM across distinct hydrologic regimes, underscoring the importance of long-term monitoring. The processing of effluent DOM by treatment wetlands represents an important, but underappreciated global service to society. Collectively, our findings will help to develop a general understanding of this important service.

## Supplementary Information

Below is the link to the electronic supplementary material.

Supplementary file1 (DOCX 381 KB)

## Data Availability

The data generated as part of this study are available in the supplementary material and within the FRDR data repository (10.20383/103.0666). All additional hydrologic, meteorological, chemical, and limnological data used here are publicly available from a previous publication (Zhu et al., 2019), the Government of Alberta, Cargill Foods Ltd., The Town of High River, or from the corresponding author (MJB) upon request.
