# Supplementary material for: Organic matter cycling in a model restored wetland receiving complex effluent
Source: Biogeochemistry. 2022 Dec 11;162(2):237–55. doi: 10.1007/s10533-022-01002-x (PMC9873720; doi:10.1007/s10533-022-01002-x)
Supplement: Supplementary file 1 — Supplementary file1 (DOCX 381 KB) [file 10533_2022_1002_MOESM1_ESM.docx]

**Supporting Information for:**

**Title:** *Organic matter cycling in a model restored wetland receiving complex effluent*

**Running Title:** *Organic effluent cycling in a restored wetland*

**Authors:** Xingzi Zhou^1^ ([xingzi.zhou@uleth.ca](mailto:xingzi.zhou@uleth.ca)), Sarah Ellen Johnston^1,2^ ([sarah.johnston3@uleth.ca](mailto:sarah.johnston3@uleth.ca); ORCID: 0000-0002-6237-0379), Matthew J. Bogard^1^ ([matthew.bogard@uleth.ca](mailto:matthew.bogard@uleth.ca); ORCID: 0000-0001-9491-0328)

**Address:** ^1^Department of Biological Sciences, University of Lethbridge, Lethbridge, AB, Canada

^2^Now at: Department of Chemistry and Biochemistry, University of Alaska Fairbanks, Fairbanks, AK, USA

**Corresponding author:** Matthew J. Bogard ([matthew.bogard@uleth.ca](mailto:matthew.bogard@uleth.ca))

**Supporting Figures:**


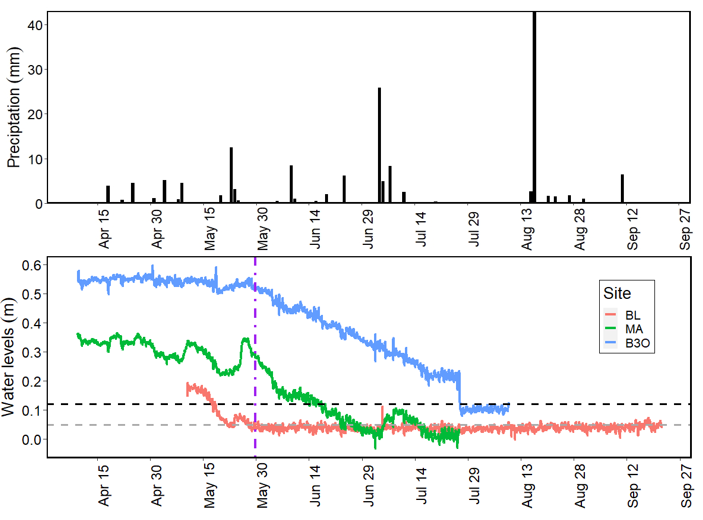


Fig. S1. Hydrology graphs at Blackie (BL), Mazeppa (MA) and Basin 3 outflow (B3O) during April to September, 2021. Daily precipitation (top panel) from Blackie AGCM station (Government of Alberta), water level (bottom panel) was collected from water level data loggers (HOBO^®^ U20L), which convert water pressure to water level at each site. B3O water level that was below black dash line (0.12 m) and BL and MA water levels that were below grey dash line (0.05 m) were below detection limits. MA had no flow since the beginning of the measurement time (April 9) and BL and B3O had no flow beyond May 19 and B3O had no flow beyond May 29 based on water depth (shown in vertical purple dashed-dotted line).


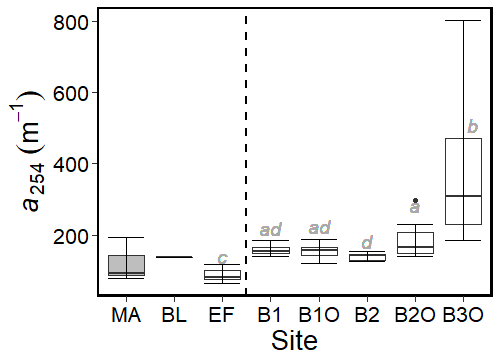


Fig. S2. Box plot of absorbance coefficients at 254 nm (*a*_254_) among sites at the Frank Lake wetland complex. All inlets, MA, BL, and EF are to the left of the dashed line and Frank Lake Basins outflows (B1O, B2O, and B3O) and spatial survey (B1 and B2) are to the right of the dashed line. ANOVA (*p* < 0.001) indicated significant differences between groups, with groupings denoted by grey, italic, lower case letters summarizing post hoc comparisons. BL and MA (grey box) were not included in the ANOVA due to only one data point being available for BL and that water from MA was stagnant and not contributing to Frank Lake.


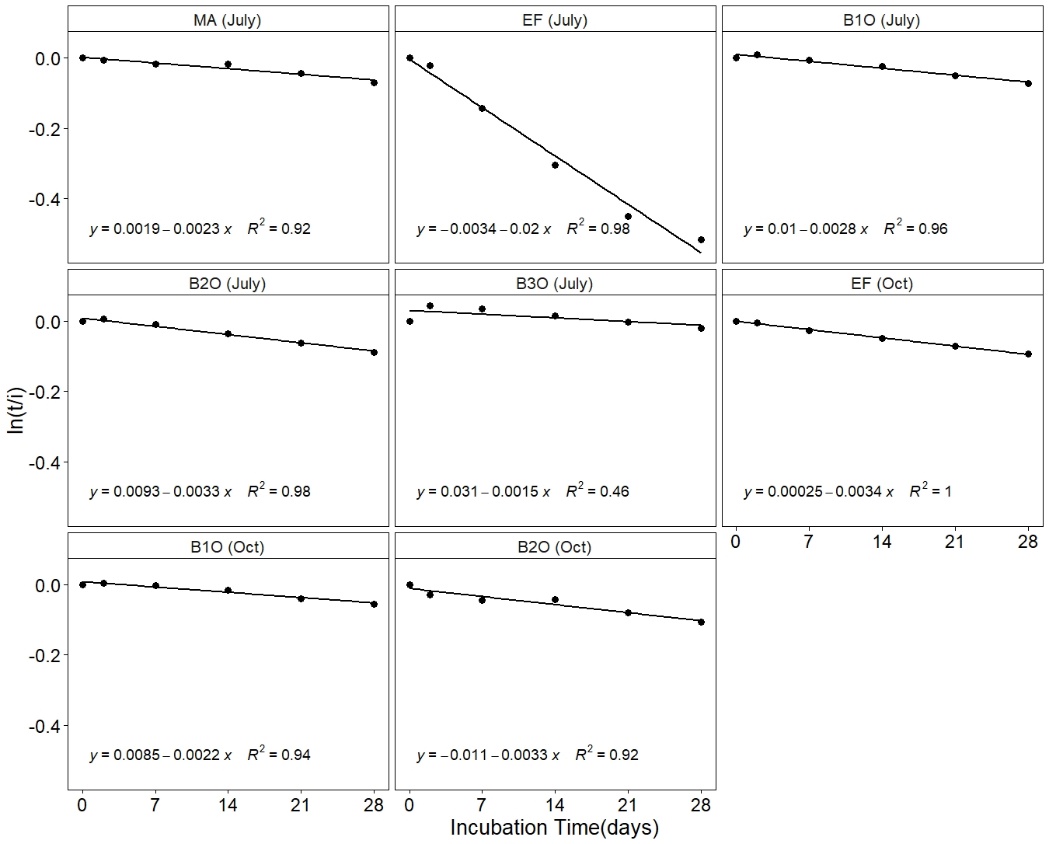


Fig. S3. Loss of DOC through time in BDOC incubations. The decomposition rate of DOC (ln(t/i)) is plotted for all sites for the July 14 sampling period (five sites for July) and the October 21 sampling period (three sites for Oct). Each point represents the mean of three subsamples.


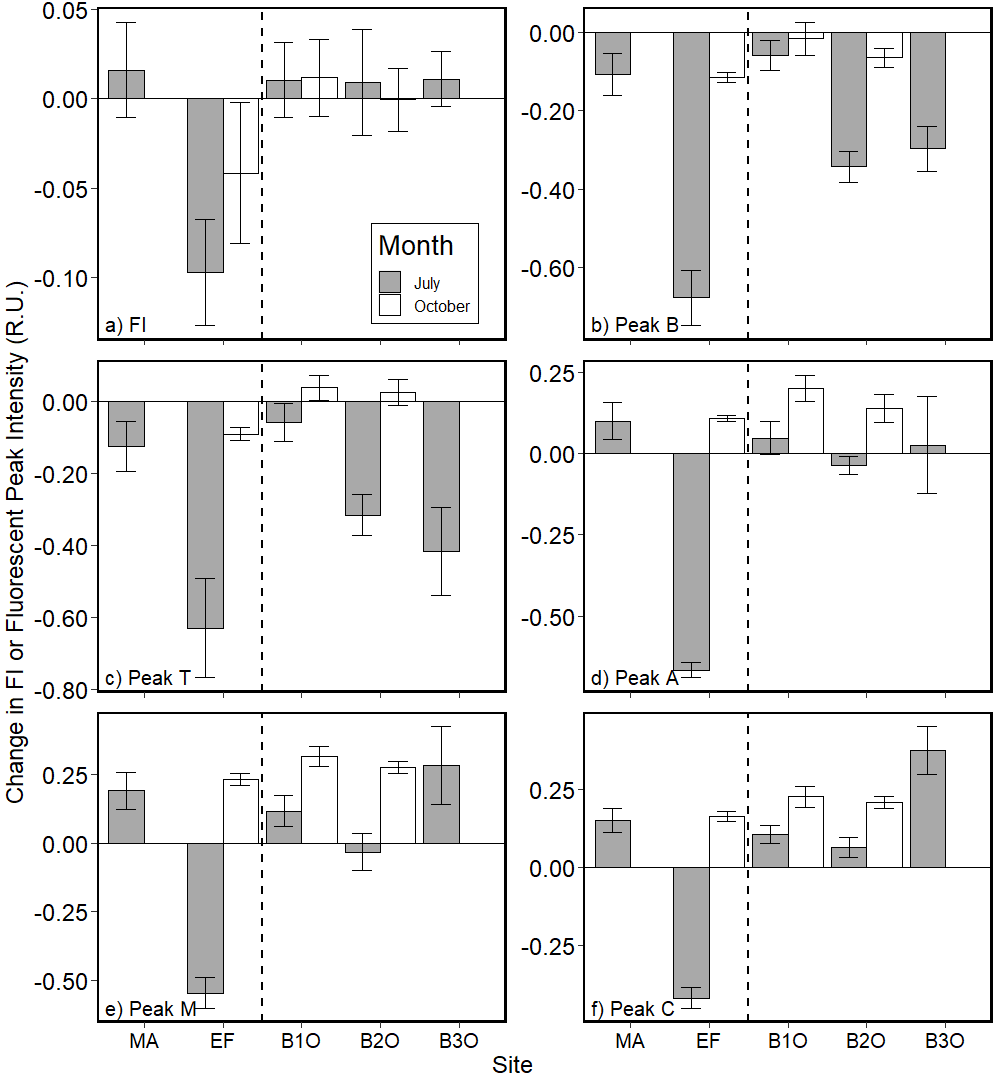


Fig. S4. The change in fluorescence index (FI) (a) and FDOM compositions (B, T, A, M, and C peaks) (b to f) in the BDOC incubations for July 14 (dark grey) and Oct 21 (white), with inlets (left of dashed line) separated from outlets (right of dashed line). These changes represent the difference between day 0 and 28. Note that MA and B30 were not flowing, and these incubations were conducted with stagnant water. Error bars represent +/- 1 S.D.


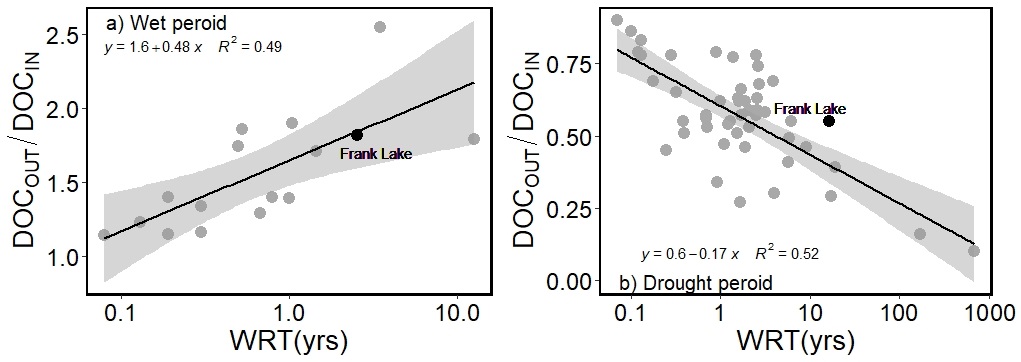


Fig. S5. The relationship between the ratio of DOC mass flux out (DOC_out_) to mass flux entering (DOC_in_) (DOC_OUT_/DOC_IN_) and water residence time (WRT) for lakes in Evans et al. (2017) (grey points) relative to Frank Lake (labelled, black points) during a wet period (2013-2015, a) and the most recent drought period (2021, b). As done in Evans et al. (2017), we present separate relationships for systems where DOC_OUT_/DOC_IN_ ≥ 1.1 (a) and those with DOC_OUT_/DOC_IN_ ≤ 0.9 (b). Linear regression (best fit) models are represented by the black line, with the 95% confidence interval represented in grey shade for relationship between DOC_OUT_/DOC_IN_ and log(WRT) lakes.

| Site | Discharge (m^3^ s^-1^) ^b^ | Water Temp.  (^o^C)^a^ | Alkalinity CaCO3 (mg L^-1^) ^a^ | Salinity (mg L^-1^) ^a^ | pH ^a^ | Specific cond.  (µS cm^-1^) ^a^ | TOC  (mg L^-1^)^a^ | TN  (mg L^-1^)^a^ | TP  (mg L^-1^)^a^ | DOC (mg L^-1^)^a^ |
| --- | --- | --- | --- | --- | --- | --- | --- | --- | --- | --- |
| Inlet |  |  |  |  |  |  |  |  |  |  |
| Blackie | 0.02 (0.07) | 9.9  (7.1) | 178.0 (88.7) | 9 (6) | 8.0 (0.4) | 1499 (1020) | 26.0 (4.8) | 2.2 (0.6) | 0.9 (0.3) | 24.4 (4.8) |
| Mazeppa | 0.03 (0.08) | 9.5  (7.8) | 188.5 (94.9) | 8 (3) | 8.0 (0.2) | 1129 (624) | 22.0 (6.9) | 1.9 (0.8) | 0.4 (0.2) | 21.9 (6.0) |
| Effluent | 0.12 (0.03) | 18.9  (5.0) | 303.0 (102.0) | 245 (131) | 7.1 (0.2) | 2330 (443) | 12.6 (3.6) | 57.4 (20.2) | 4.4 (1.1) | 11.5 (3.0) |
| Outlet |  |  |  |  |  |  |  |  |  |  |
| Basin 1 outflow | 0.21 (0.40) | 13.6  (6.6) | 307.5 (88.0) | 112 (47) | 8.7 (0.6) | 1703 (538) | 23.6 (6.3) | 7.2 (4.0) | 2.2 (0.9) | 23.5 (5.4) |
| Basin 2 outflow | 0.25 (0.44) | 13.5  (7.0) | 404.3 (87.1) | 189 (142) | 8.7 (0.5) | 2011 (416) | 24.9 (4.4) | 4.9 (2.6) | 2.2 (0.9) | 25.7 (4.8) |
| Basin 3 outflow | 0.23 (0.32) | 14.3  (6.9) | 471.3 (180.7) | 211 (126) | 8.3 (0.5) | 2430 (933) | 32.9 (9.5) | 3.8 (1.5) | 2.6 (1.1) | 32.0 (9.0) |

Table S1. Physical and chemical features of sampling sites in Frank Lake. Mean of measurements are included and standard deviation is shown (in brackets) if applicable*.

*Mean values calculated by using all data from 2012 to 2018 that are available.

^a^ Data from Alberta Environment and Parks.

^b^ Data from Alberta Environment and Parks and Zhu et al. (2019).

| Component | Ex max (nm) | Em max (nm) | Potential sources | Relative contribution at each site (%) | | | | |
| --- | --- | --- | --- | --- | --- | --- | --- | --- |
|  |  |  |  | MA | EF | B1O | B2O | B3O |
| C1 | 265 | 422 | Terrestrial humic-like ^a, b^ | 30.0 | 11.7 | 25.9 | 27.0 | 31.1 |
|  |  |  |  |  |  |  |  |  |
| C2 | 335 | 416 | Wastewater related or from nutrient rich environment ^c, d^ | 19.7 | 26.8 | 18.4 | 18.3 | 20.1 |
|  |  |  |  |  |  |  |  |  |
| C3 | 365 | 478 | Mixture of peak A and C ^e^ | 18.9 | 14.8 | 13.4 | 13.7 | 16.2 |
|  |  |  |  |  |  |  |  |  |
| C4 | 305 | 372 | Microbial humic-like ^f^ | 15.6 | 21.8 | 20.0 | 19.4 | 17.3 |
|  |  |  |  |  |  |  |  |  |
| C5 | 280 | 328 | Autochthonous protein-like, tryptophan-like (peak T) ^a, g^ | 15.8 | 24.9 | 22.2 | 21.7 | 15.4 |

Table S2. The characteristics of the five components in the PARAFAC model and their relative abundance at each site.

Literature sources: ^a^ Coble (2007), ^b^ Wünsch et al. (2017), ^c^ Jutaporn et al. (2020), ^d^ Murphy et al. (2011),

^e^ Yamashita et al. (2011), ^f^ DeFrancesco and Guéguen (2021), and ^g^ Osburn et al. (2011).

| Site | Initial DOC (mg L^-1^) | Final DOC (mg L^-1^) | *a*_254_ (m^-1^) | *S*_R_ | SUVA_254_ (L mg C ^-1^ m^-1^) | | FI | A:T | | *k* (day^-1^) | | t_1/2_ (days) | |
| --- | --- | --- | --- | --- | --- | --- | --- | --- | --- | --- | --- | --- | --- |
| July |  |  |  |  |  | |  |  | |  | |  | |
| MA | 31.3 (0.6) | 29.2 (0.4) | 192 (5) | 0.98 (0.05) | 2.73 (0.05) | | 1.42 (0.01) | 2.27 (0.13) | | 0.0023 | | 301 | |
| EF | 20.8 (0.6) | 12.4 (0.1) | 90 (5) | 0.43 (0.10) | 2.41 (0.40) | | 1.91 (0.04) | 0.86 (0.06) | | 0.02 | | 35 | |
| B1O | 32.7 (0.3) | 30.4 (0.2) | 136 (2) | 1.00 (0.18) | 1.85 (0.05) | | 1.48 (0.01) | 1.67 (0.06) | | 0.0028 | | 248 | |
| B2O | 39.5 (0.7) | 36.2 (0.1) | 191 (6) | 1.07 (0.09) | 2.17 (0.03) | | 1.48 (0.01) | 1.84 (0.18) | | 0.0033 | | 210 | |
| B3O | 109.3 (0.9) | 107.3 (2.8) | 498 (12) | 1.04 (0.05) | 1.95 (0.02) | | 1.44 (0.01) | 2.42 (0.17) | | 0.0015 | | 462 | |
| October |  |  |  |  |  | |  |  | |  | |  | |
| EF | 12.4 (0.1) | 11.3 (0.2) | 76 (1) | 0.41 (0.06) | | 2.76 (0.07) | 1.87 (0.02) | | 1.02 (0.05) | | 0.0034 | | 204 |
| B1O | 37.9 (0.4) | 35.9 (0.2) | 160 (2) | 0.89 (0.09) | | 1.87 (0.05) | 1.47 (0.02) | | 1.62 (0.11) | | 0.0022 | | 315 |
| B2O | 41.6 (2.8) | 37.4 (0.3) | 155 (2) | 1.04 (0.15) | | 1.71 (0.07) | 1.47 (0.02) | | 1.58 (0.11) | | 0.0033 | | 210 |

Table S3. Summary of DOC concentration and optical properties for the 28-day BDOC experiments. Standard deviation in brackets if applicable.
